# Taxonomic studies of the *Lygephila lubrica* (Freyer, 1842) species complex with notes on other species in the genus (Lepidoptera, Erebidae, Toxocampinae)

**DOI:** 10.3897/zookeys.452.8152

**Published:** 2014-11-05

**Authors:** Oleg Pekarsky

**Affiliations:** 1 H-1068, Budapest, Felsőerdősor u. 16–18, Hungary

**Keywords:** Lepidoptera, Erebidae, Toxocampinae, *Lygephila lubrica* species complex, *Lygephila mirabilis*, *Lygephila lupina*, vesica structure

## Abstract

The taxa of the *Lygephila lubrica* (Freyer, 1846) species complex are revised. The genital features of all known taxa are described and illustrated, with special reference to the structure of the vesica. Genitalia of *Lygephila lubrica* from different places in Russia, Central Asia and China are studied, illustrated and compared with different Mongolian populations. *Lygephila kazachkaratavika*, described as a subspecies, is raised to a species level, **stat. n.** Neotypes of *Lygephila lubrosa* (Staudinger, 1901), *Lygephila lubrosa kazachkaratavika* Stshetkin YuL & Stshetkin YuYu, 1994 [1997] and *Lygephila lubrosa orbonaria* Stshetkin YuL & Stshetkin YuYu, 1994 [1997] are designated. The female genitalia of the type of *Lygephila lupina* (Graeser, 1890) is described and illustrated for the first time, and *Lygephila mirabilis* (Bryk, 1948) treated here as a junior subjective synonym, **syn. n.**

## Introduction

This paper is dedicated to clarify the taxonomic status of the taxa in the *Lygephila lubrica* species group, which is proved to contain more than a single species. Special attention was paid to revising the poorly-known taxa described from Central Asia and the identity of the historical names that have been used confusingly in the literature. The examined material is considered as representative for the entire area of the species complex, including all available types preserved in the collections of Püngeler, Staudinger, Bang-Haas, and Stshetkin. Neotypes are designated when required by the taxonomic results.

## Materials and methods

Male and female genitalia were dissected and mounted in Euparal on glass sides. Photos of genitalia were made by Svitlana Pekarska using a Nikon SMZ745T microscope and Moticam 2500 camera. Photos of imagines where taken by the author using a Nikon D3000/Sigma 105, f/2.8 camera.

**Abbreviations:** HNHM = Hungarian Natural History Museum Budapest (Hungary); IZIP = Institute of Zoology and Parasitology, Tajik Academy of Sciences Dushanbe (Tajikistan); MA = Matov Alexey, St. Petersburg (Russia); MNHU = Museum für Naturkunde der Humboldt-Universität zu Berlin (Germany); NHMW = Naturhistorisches Museum Wien (Vienna, Austria); ZISP = Zoological Institute, Russian Academy of Sciences St. Petersburg (Russia); ZFMK = Zoologisches Forschungsinstitut und Museum Alexander Koenig, Bonn; ZSM = Zoologische Staatssammlung München; AV = Anton Volynkin (Barnaul, Russia); GB = Gottfried Behounek (Grafing, Germany); JB = János Babics (Budapest, Hungary); OP = Oleg Pekarsky (Budapest, Hungary); LR = László Ronkay (Budapest, Hungary); WB = Wiltshire Berlin (slide made by Edward P. Wiltshire in the collection of MNHU).

## Systematic accounts

### Description of the *Lygephila lubrica* species complex

Head and body brownish grey with frons and collar chocolate brown. Forewing broad, apex less pointed than in the *Lygephila lusoria* group (Babics and Ronkay 2009, Pekarsky 2013), ground colour brownish grey or unicolorous grey with traceable crosslines; orbicular stigma as a small white dot, reniform stigma large, approximately triangular, dark brown; hindwing ground color varies from brown and greyish brown to yellowish or intensive ochreous, discal spot visible only on underside, transverse line distinct, marginal band conspicuously dark. Male genitalia of *Lygephila lubrica* nearly symmetrical; clasping apparatus of other species slightly asymmetrical (right valva narrower with longer ampulla), uncus well developed, long, strong, its distal half broadened with acute tip; valva margins more or less parallel with rounded apex; clasper long, sclerotized, digitiform, located subapically; aedeagus cylindrical, long, straight; vesica globular, membranous, multidiverticulate (six or seven diverticula various in shape and size), terminal tube long, membranous; ostium bursae large; antrum sclerotized, funnel shaped with considerable cleft in middle of posterior margin; ductus bursae small, corpus bursae membranous, elliptical or ovoid.

#### Lygephila lubrica lubrica

Taxon classificationAnimaliaLepidopteraErebidae

(Freyer, 1846)

Figs 1–10

Ophiusa lubrica Freyer, 1846, Neuere Beiträge zur Schmetterlingskunde mit Abbildungen nach der Natur. 6: 7, Tab. 483, fig. 4. (TL: not given)
Lygephila lubrica sublubrica (Staudinger, 1896);
Toxocampa lubrica var. sublubrica Staudinger, 1896, Deutsche Entomologische Zeitschrift Iris 8: 271. (TL: [Mongolia, Uliastai], Uliassutai)

##### Type material examined.

*Lygephila lubrica sublubrica* (Staudinger, 1896), **Type** ♂, [Mongolia, Uliastai], Uliassataj, slide No. WB12 (coll. MNHU).

##### Additional material examined.

2 ♂♂ & 1 ♀, Russia, C Tuva, W of Ujukskyi Mts, Kamennyi riv. valley, h=800–1000 m, 11–20.07.2003, leg. S. Vaschenko, slide Nos: OP1955m, OP2438m, OP1956f (coll. O. Pekarsky); 1 ♂, Russia, Altai Mts, 700 m, Kupchegen, 23–25.VII.2002, leg. Hácz & Juhász, slide No. OP1962m (coll. O. Pekarsky); 1 ♂, Russia, NW Altai Mts, Tigireksky ridge, slide No. AV0907 (coll. A. Volynkin); 1 ♂ Russia, Altai rep., Aktash, 1400 m, 12–14.VIII.2010, leg. R. Yakovlev, slide No. OP2439m (coll. O. Pekarsky); 1 ♀, Russia, SE Altai Mts, Aktash vill., slide No. AV0906 (coll. A. Volynkin); 1 ♂, Mongolia, Central aim., Nr. 1148, leg. Z. Kaszab, slide No. LR1401m (coll. HNHM); 1 ♂, Mongolia, Chövsgöl aimak, Nr. 1128, leg. Z. Kaszab, slide No. LR1402m (coll. HNHM); 1 ♀, Mongolia, Central aimak, 26 km O von Somon Lun, 1180 m, Nr. 260, 3.VIII.1964, leg. Z. Kaszab, slide No. OP2010f (coll. HNHM); 1 ♂ & 2 ♀♀, Mongolia, Selenga aimak, Orhon v., Sir Orhon, 715 m, N49°08'956", E105°15'099", 3–4.07.2004, leg. K. Gaskó, slide Nos: OP2296m, OP2295f, OP2297f (coll. P. Gyulai); 1 ♂, [Kazakhstan], Russia, Uralsk, 1937.VII., ex coll. Velez, slide No. LR1403m, (coll. HNHM); 1 ♂ & 1 ♀, [Kazakhstan], Uralsk, slide Nos: Hacker2536m/ZSM2510m, Hacker2334f/ZSM2508f (coll. ZSM); 1 ♂ & 1 ♀, Russia, S Ural, Orenburg reg., Donskoe vill., Verbljushka Mt., 25–29.6.2009, leg. L. Srnka, slide Nos: OP2124m, OP2125f (coll. O. Pekarsky); 4 ♂♂ & 1 ♀, Russia, Bashkortostan, Yantysh vill., 29–31.VII.2011 slide Nos: OP2005m, OP2007m, OP2440m, OP2441m, OP2006f (coll. O. Pekarsky); 1 ♂ & 1 ♀, Russia, Kabardino-Balkaria, C Caucasus Mts, Bydyk, 1250m, 18.7.2012, leg. L. Srnka, slide Nos: OP2151m, OP2152f (coll. O. Pekarsky); 1 ♀, Kasakhstan, 40 km W Ust Kamenogorsk, Kalbinski Altai, Monastyri, 600 m, 06.08.1994, leg. Lukhtanov, slide No. OP2013f (coll. P. Gyulai); 1 ♂, Kazakhstan, Boro-Khoro Mts, 30km N of Panfilov, (20 km from Chinese border), N44°29'765" E80°03'848", 1830 m, 30.06.2010, leg. S.K. Korb, slide No. OP2083m (coll. O. Pekarsky); 1 ♂, Kirgizstan, Inner Tjan-Shan, Min-Kush circ., 2300 m, 2.08.2000, leg. I. Pljushtch, slide No. OP2004m (coll. O. Pekarsky); 1 ♂ & 1 ♀, Kyrgyzstan, Naryn reg., Kekemeren river, n., Sarykamysh, 1400 m, 6–8.07.1996, leg. V.A. Lukhtanov, slide Nos: OP2015m, OP2016f (coll. P. Gyulai); 1 ♂, [Kyrhyzstan], Issykkul, Tianschan, 949, ex coll. Kotzsch, slide No. OP2426m (coll. ZFMK); 2 ♀♀, China, Xinyiang [Xinjiang] – Uygur, Boro Horo Shan, Balguntay city, 2000 m, 13.7.1996, leg. Nykl, slide Nos: OP2011f, OP2012f (coll. P. Gyulai); 1 ♂, China, Boro Boro shan, Balguntay city, 2000 m, 13.7.1996, slide No. OP2289m (coll. P. Gyulai); 1 ♀, Aksu Bakalik, Anf. VI.1912, ex coll. Rückbeil, slide No. OP2339f (coll. ZSM); 1 ♂, Aksu Bakalik, Anf. VI.1912, ex coll. Rückbeil, slide No. OP2338m (coll. ZSM); 1 ♂, [China], Aksu, [19]11, 225, ZFMK76/64 Boppard, slide No. OP2427m (coll. ZFMK); 1 ♂, Mongolia, Uliasutai, slide No. 0326Matov (coll. ZISP); 1 ♂, [Mongolia], Uliassatai, 946, ex coll. Kotzsch, 8/57, slide No. OP2428m (coll. ZFMK); 1 ♂ & 1 ♀, SW Mongolia, Hovd aimak, Bodonchijn-Gol basin, Hundij-Gol riv. valley, 1600 m, 46°06’N; 92°30’E, 3.vii.2010, leg. E. Guskova & R. Yakovlev, slide Nos: OP1957m, OP1958f (coll. O. Pekarsky); 2 ♂♂, W Mongolia, Hovd aimak, near Erdene-Buren somon, h=1 400 m, 04.07.2007, leg. Yakovlev R.V. & Guskova E.V., slide Nos: AV0283, AV0285 (coll. A. Volynkin); 1 ♂ & 1 ♀, Mongolia, Hovd Aimak, Altaj Mts, 10 km NE of Dott, 2000 m, 10.08.1996, leg. S. Farkas & I.Zs. Tóth, slide No. OP2290m, OP2291f (coll. P. Gyulai); 2 ♂♂ & 1 ♀, W Mongolia, Hovd aimak, near Erdene-Buren-Somon, 1400 m, 1.07.2010, 2500–2850 m, leg. R. Yakovlev, E. Guskova, slide Nos: OP2350m, OP2351m, OP2352f (coll. O. Pekarsky); 1 ♂, Mongolia, Bulgan aimak, 54 km W of Erdenecant, 1260 m, 104°05’E 47°05’N, 22.07.1987, leg. L. Peregovits, M. Hreblay & T. Stéger, slide No. OP2008m (coll. HNHM); 1 ♂, Mongolia, [Khentii] Chentaj aimak, Tsenkher-Mandal, Modoto, 1600–1800 m, 9–14.07.1984, leg. K. Cerny, slide No. GB2550m, (coll. G. Behounek); 1 ♂, [Russia], Yakovlevka Spas. u., Ussur. kr., 12.VIII.[1]926, [leg.] D’iakonv Filip’ev (in russian), slide No. 0330Matov, (coll. ZISP); 1 ♀, Russia, Primorsky ter., Lesozavodsk reg., Innokentievka, 26–30.VIII.[19]94, slide No. OP2298f (coll. P. Gyulai); 1 ♀, [China], Mien-shan (Prov. Shansi), Obere Höhe ca. 2000 m, 2.8.1937. [leg.] H. Höne, slide No. OP2423f, 2 ♂♂, 9.8.1937, slide Nos: OP2421m, OP2425m, 1 ♂, 13.8.1937, slide No. OP2422m (coll. ZFMK); 1 ♀, [China], Tapaishan im Tsinling, Sued-Shensi, Ca. 3000 m, 17.6.1936, [leg.] H. Höne, slide No. OP2424f (coll. ZFMK).

##### Taxonomy.

*Lygephila lubrica* was described in 1846 by Freyer in the genus *Ophiusa*. The exact type locality was not given in the original paper and also there was no information about the types. In 1896, Staudinger supposed during the description of Toxocampa lubrica var. sublubrica, that *Ophiusa lubrica* was described by Freyer from Altai: «Freyer sagt von seiner *Lubrica* nur, dass er sie von Kindermann erhielt; es muss sicher die von diesem Sammler im Altai gefundene Art sein, von der ich drei Stücke aus Lederer’s Sammlung besitze». Based on this assumption the type locality of *Lygephila lubrica* is most probably “Russian Altai” near Ust-Bukhtarminsk settlement (not existing now), which was located near the junction of the Bukhtarma and Irtysh rivers in the modern territory of Kazakhstan. Staudinger & Wocke (1871) placed this species in the genus *Toxocampa*, and later Staudinger (1896) described a variation named as *sublubrica* from Uliastai on the western edge of Khangai Mountains in the western part of Mongolia. The type specimen of *sublubrica* was not found in the collection of MNHU in Berlin however the genitalia slide made by Edward Wiltshire is in the museum (genitalia slide collection, Figs 27, 28). The current combination – *Lygephila lubrica* – occurs first in Sheljuzhko (1967) and later in Ronkay (1983). The taxon *sublubrica* is considered as a subspecies of *Lygephila lubrica* in these two works. Poole (1989) incorrectly treated *Lygephila lubrica* (Freyer, 1842) as a new combination, and listed Toxocampa lubrica var. sublubrica Staudinger, 1896 and Toxocampa lubrica var. lubrosa Staudinger, 1901, and have been listed as such in subsequent works (e.g., Goater et al. 2003; Kononenko 2010).

##### Diagnosis.

The main external distinctive feature of the species is the brownish-grey ground colour of forewings and hindwings. *Lygephila lubrica* differs from the externally somewhat similar *Lygephila lubrosa* by its characteristic brownish-grey ground color of the forewings; from *Lygephila kazachkaratavika* by more unicolorous forewings with a less-developed pattern; and from both related species by its brownish hindwings, which are generally ochreous in the two latter species. The differences in the genitalia structures among the three similar species are easily recognisable in both sexes. In males, the uncus dilation in *Lygephila lubrica* is wider than in *Lygephila lubrosa*, but narrower than in *Lygephila kazachkaratavika*, and the ampulla is more proximal, closer to the middle of the valve, than in the two other species; in the females, the cleft on the posterior margin of the antrum is U-shaped or V-shaped in *Lygephila lubrica*, whereas in *Lygephila lubrosa* it is evenly concave; in *Lygephila kazachkaratavika* the ostium cleft is deep, narrow, slit-like.

##### Description.

Wingspan 37–50 mm, on average 42–48 mm. Head and body brownish grey; collar dark chocolate brown. Forewing brownish grey, sometimes dark brown; subbasal line indistinct; antemedial line arched, consisting of two elongated patches; medial fascia diffuse, wide and waved, with two costal patches; reniform stigma approximately triangular, dark brown, sometimes with sharp extension at inner corner and with satellite streak-like spots on outer margin; orbicular stigma as small white dot; postmedial line distinct; subterminal line with light fascia; terminal line a black sinuous stripe. Hindwing varies from brown to greyish brown; transverse line distinct; narrow discal spot present on underside; outer dark third with defuse inner margin; fringes as ground color.

**Male genitalia** (Figs 21–32, 39–41). Uncus with short stem and dilated distal two thirds, apex with fine tip, anal tube membranous with characteristic oval hardening of tissue - scaphial crown on scaphium and sclerotized fig on subscaphium; valva elongated, relatively wide with parallel margins, valval apex rounded; clasper digitiform, slightly curved towards costa, situated rather far from apex. Aedeagus straight, long, tubular. Vesica globular, multidiverticulate, membranous; 1^st^ subbasal diverticulum small, adjacent to 2^nd^ terminal diverticulum; medial diverticulum large, tapering, with medium-large oblong chamber at base; 1^st^ terminal diverticulum large, more or less wedge shaped with one part densely scobinated and membranous, cauliflower-like, opposite part bears numerous small pockets; 2^nd^ terminal diverticulum tubular, scobinated on top; 3^rd^ terminal diverticulum irregular shaped with large rectangular scobinated basal part and membranous cylindrical extension; 4^th^ terminal diverticulum medium sized, situated between 1^st^ and 3^rd^ medial diverticulum; 2^nd^ subbasal diverticulum small, tubular, sometimes chili-pepper-like (Fig. 41), terminal tube membranous, as long as aedeagus, opening point of terminal tube located subbasally near carina.

**Female genitalia** (Figs 46–57). Ovipositor relatively large, broad, papillae anales hairy with long setae on apical edges. Apophyses anteriores stout, apophyses posteriores thin, longer than apophyses anteriores. Ostium broad, antrum tapering, funnel shaped, posterior margin incised producing large U-shaped cleft; ductus bursae small, inflated with ventral sclerotized ribbon; appendix bursae small; corpus bursae membranous, ovoid.

**Figures 1–10.**
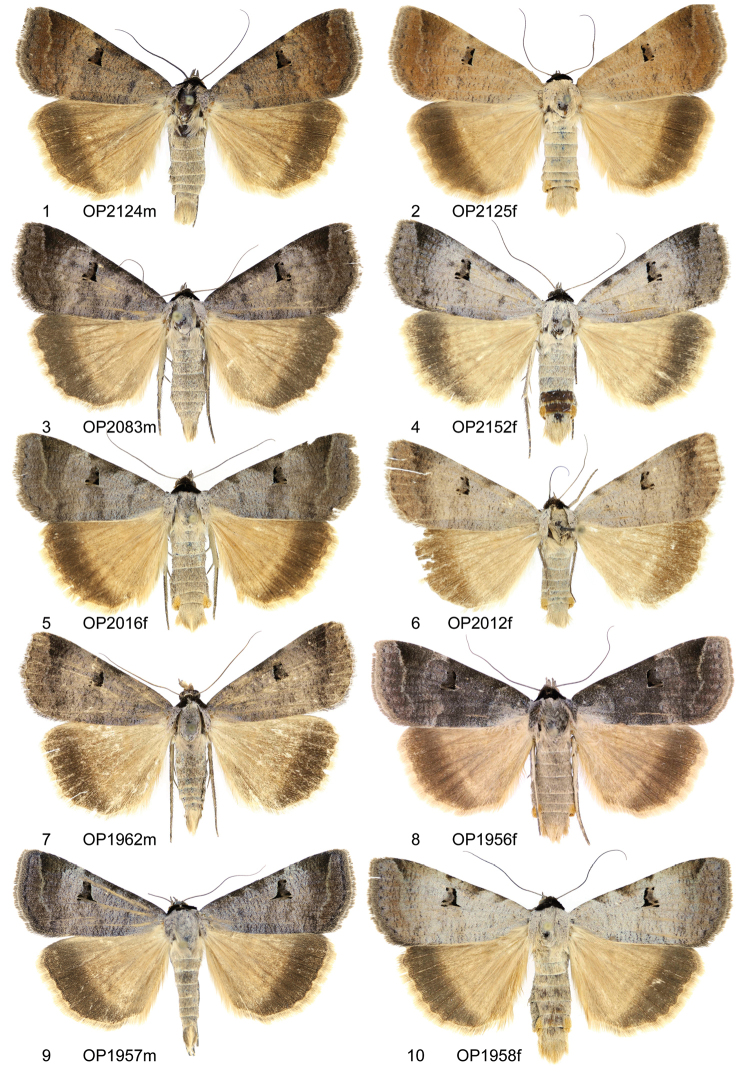
Adults. *Lygephila lubrica* **1** ♂, Russia, Orenburg **2** ♀, Russia, Orenburg **3** ♂, Kazakhstan, Boro-Khoro Mts **4** ♀, Kabardino-Balkaria, Bydyk **5** ♀, Kyrgyzstan, Naryn reg. **6** ♀, China, Xinyiang – Uygur **7** ♂, Russia, Altai, Kupchegen **8** ♀, Russia, Tuva **9** ♂, SW Mongolia, Hovd aimak **10** ♀, SW Mongolia, Hovd aimak.

##### Distribution.

Siberian. Distributed from Zaporozhie region of Ukraine to Rostov, Samara, Povolzhie regions to Ural of Russia through Kazakhstan, Russian Altai and northern Mongolia.

#### Lygephila lubrosa lubrosa

Taxon classificationAnimaliaLepidopteraErebidae

(Staudinger, 1901)

Figs 17 , 18

Toxocampa lubrica var. lubrosa Staudinger, 1901, Catalog der Lepidopteren des Palaearctischen Faunengebietes. I: 252. (TL: [Kazakhstan], Ili, [Kyrgyzstan, Issyk Kul], “Iss. K.”)

##### Type material examined.

**Neotype** (here designated) male, Kazakhstan, Ili river valley near bridge 23,4 km asimut 222 from Koktal, 600 m, N43°58'004", E79°35'905", 04.07.2010, leg. S.K. Korb, slide No. OP2082m (coll. O. Pekarsky, deposited in HNHM Budapest).

##### Additional material examined.

1 ♂, with same data as neotype; 1 ♂ & 1 ♀, Kazakhstan, Ili river valley near Koktal, 506 m, N43°57'57.50", E79°36'1.06", 03.07.2010, leg. S.K. Korb, slide No. OP2489f (coll. O. Pekarsky); 1 ♀, [Kazakhstan], Syr-Daria, Baigacum, Koshantschikoff, 23.6.1913, 4/7, ex. coll. Püngeler, slide No. OP1979f (coll. MNHU); 1 ♂, [Kazakhstan], Aj-Darle, Syr-Darja, 25.V.1909, leg. Koshantshikoff, slide No. 0325Matov (coll. ZISP).

##### Taxonomy.

Described by Staudinger in 1901 as a variation of *Lygephila lubrica*; with the type locality mentioned as [Kazakhstan], Ili [river] and [Kyrgyzstan], Issyk Kul [lake]. The original description stated that the forewings are pale grey (“cinereo-griseis”) without dark outer part, and that the hindwings are ochreous with broad marginal fascia. This description corresponds exactly with the external appearance of the moths from Ili river in Kazakhstan, therefore the neotype is designated from this area. Moths from Issyk Kul show, however, marked differences in habitus, especially the brown coloration of most parts of the forewings. These two taxa are different in genital structures of both sexes, which are discussed in detail under *Lygephila kazachkaratavika*. Starting from the 1980’s, Stshetkin YuL treated *Lygephila lubrosa* in his publications as a distinct species (Stshetkin et al. 1988, Stshetkin 1991). The explanation of this act was given only in 1994 [1997] by Stshetkin YuL & Stshetkin YuYu. Their argumentation was based only on the original description of *Lygephila lubrosa*, but neither the type material nor the genitalia of the syntypes were studied. Unfortunately, the authors evidently failed in their taxonomic interpretation of the species complex. They were correct to suppose *Lygephila lubrosa* Staudinger, 1901 is a separate taxon different from *Lygephila lubrica*, but they failed to define this taxon, and did not recognize that the yellowish hindwinged populations include two different species.

The main fault of the Stshetkins’ work is the lack of definition of *Lygephila lubrosa* Staudinger, 1901. In their article they provided the following description of the genitalia of *Lygephila lubrosa*: “Гениталии самца симметричные. Ункус слабо изогнутый, расширенный в средней части, заостренный. Вальвы удлинённые с немного выпуклыми дорзальными и вентральными краями. Вершинный отросток класпера пальцеобразный, длиннее, чем у *Lygephila lubrica*; его конец находится близ дорзального края вальвы (у *Lygephila lubrica* он далеко не достигает края). Конец вальвы от основания этого отростка до его заднего конца заметно короче, чем у *Lygephila lubrica*. В оральной трети длины вальвы продольная хитинизированная складка класпера, направляясь орально, плавно прогибается несколько к вентральному краю вальвы и при этом не образует резкого угла с бугорком-гарпой, имеющегося у *Lygephila lubrica* Frr. Нижняя фультура под эдеагусом без особого изгиба прямо идет в сторону саккуса, как у *Lygephila lusoria* L.” The translation of this text is as follows: “The male genitalia are symmetrical. Uncus slightly curved, dilated in the middle part, pointed. Valva elongated with slightly convex dorsal and ventral edges. Apex of clasper digitiform, longer than that of *Lygephila lubrica*; its end close to the dorsal margin of the valva (as for *Lygephila lubrica*, the latter is far from reaching the margin). The end of the valva from the base of the clasper till its back end is noticeably longer than that of *Lygephila lubrica*. In the oral [basal] third of the valva, the longitudinal chitinized fold of the clasper is directed orally [basally] and is slightly curved towards the ventral margin of the valva without forming an abrupt angle with the hump-harpe, which is typical for *Lygephila lubrica* Frr. Lower fultura [juxta] under aedeagus almost straight and directed towards the saccus as *Lygephila lusoria* L.” This description is contradictory as it includes characteristics of both yellow hindwinged species occurring in Central Asia. To be precise, “Uncus dilated in the middle part” is only typical for the moth (*Lygephila kazachkaratavika*) from Kyrgyzstan (lake Issyk Kul) and Kazakhstan (city of Kizilorda and station Baigacum on the river Syr Darja) (Figs 37, 38); “Apex of clasper digitiform and longer than that of *Lygephila lubrica*; its end close to the dorsal margin of the valva” is only typical for the moths (*Lygephila lubrosa*) from Kazakhstan (river Ili) and Tajikistan (river Pianj) (Figs 33–36). The female genitalia are described as follows: “В гениталиях самки копулятивная сумка мешковидная и вместе с едва заметным бугорком-буллой вся перепончатая (у *Lygephila lubrica* булла конусовидная, хитинизированная, как и весь проток и часть сумки). Проток сумки значительно короче, его оральная часть перепончатая”. The translation is as follows: “In the female genitalia the copulative pouch [corpus bursae] is saccular and all membranous along with a barely noticeable bulla (while the bulla of *Lygephila lubrica* is conical and chitinized as well as the whole antrum and part of the pouch [corpus bursae]). The antrum is significantly shorter with membranous oral [basal] part.” The characteristics mentioned as “the antrum is significantly shorter than that of *Lygephila lubrica*” partially corresponds to the moths from the Ili region. However, it is not diagnostic because in many specimens of *Lygephila lubrica* the antrum has the same length. The antrum of the moths (*Lygephila kazachkaratavika*) from the Issyk Kul region and the river Syr-Darja is one and a half times longer than that of *Lygephila lubrica* and two times longer than that of the moths from the valley of the river Ili (*Lygephila lubrosa*). The other characteristics mentioned by the authors are general, non-autapomorphic and unsuitable for determination.

In the same work the authors described two subspecies of *Lygephila lubrosa* on the basis of external characteristics, admitting that the two subspecies do not differ in genitalia structure from the nominotypical subspecies. However, the moths from the Kazakh Karatau, station Balamurum collected by V. Kozhantshikov in 1909 (*Lygephila kazachkaratavika*) differ significantly in their genitalia structure from the moths from the valleys of the river Ili (*Lygephila lubrosa lubrosa*) and the river Pianj (*Lygephila lubrosa orbonaria*). All above-mentioned data prove that the authors did not consider the subject of their research, which caused unsatisfactory results and added further difficulties for the clarification of this species-complex. A further difficulty is that the authors did not define holotypes or paratypes (or simply type series) for the newly described taxa. According to the information from the museum curators of ZISP and IZIP, they do not possess the aforesaid type specimens with the corresponding type labels.

In order to correctly identify the taxa of this species complex, in view of complexity of the current taxonomic situation, and to give an accurate definition of *Lygephila lubrosa*, it is necessary to designate a neotype of this taxon.

##### Diagnosis.

Easily distinguishable from all other members of the species group by its unicolorous grey forewings. Comparing the genital structures of the taxa of the group, it differs from all related species by the narrow uncus without a real dilatation (only some slight thickening is present), the wide valva, and the subapically located, strong clasper with its tip reaching the valval edge (males); and by the funnel-shaped antrum bent dorsally at 45 degrees, being a unique female character for the whole genus (Fig. 60).

##### Description.

Wingspan 42–46 mm. Head and body grey with some brownish scales; collar chocolate brown. Forewing almost unicolorous, wing pattern poorly developed; subbasal line hardly traceable, represented by groups of dark scales on veins; antemedial line semicircular; medial shade not expressed; reniform stigma small, indistinct, with one or two black dots basad; orbicular stigma small dot-like; postmedial and subterminal lines distinct; terminal line a row of black dots on veins. Hindwing pale ochreous; transverse line not discernible; outer dark third with sharply defined inner margin; fringes ochreous.

**Male genitalia** (Figs 33, 34, 44, 45). Uncus long, stout, slightly thickened medially with skewed fine tip, anal tube membranous with oval hardening of tissue - scaphial crown on scaphium and sclerotized fig on subscaphium; valva elongated, wide, with parallel margins in the middle, valval apex rounded; clasper digitiform, strong, thickened with wide base, placed subapically, asymmetrical, left one shorter than the right one, both almost reach valval costa. Aedeagus tubular with narrow, long, acute sclerotised lamina on ventral side of carina. Vesica globular, multidiverticulate, membranous; basal diverticulum small; medial diverticulum large with small lateral hemispherical bulging; 1^st^ terminal diverticulum large, more or less wedge shaped, membranous with cauliflower-like part bearing numerous small pockets; 2^nd^ terminal diverticulum large, cone shaped, scobinated on top; 3^rd^ terminal diverticulum medium-sized, bifurcated, Y-shaped; 4^th^ terminal diverticulum large, bilobate, located oppositely to the 3^rd^ medial diverticulum; terminal tube membranous as long as aedeagus, opening point of terminal tube located near to carina.

**Female genitalia** (Figs 58–61). Ovipositor relatively large, broad, papillae anales hairy with long setae on apical edges. Apophyses anteriores stout, apophyses posteriores thin, longer than apophyses anteriores. Antrum funnel shaped, bent dorsally at 45 degrees, boomerang shaped from lateral view; ostium bursae broad, posterior margin U-shaped; ductus bursae medium sized; appendix bursae small; corpus bursae membranous, bevelled oval.

**Figures 11–20.**
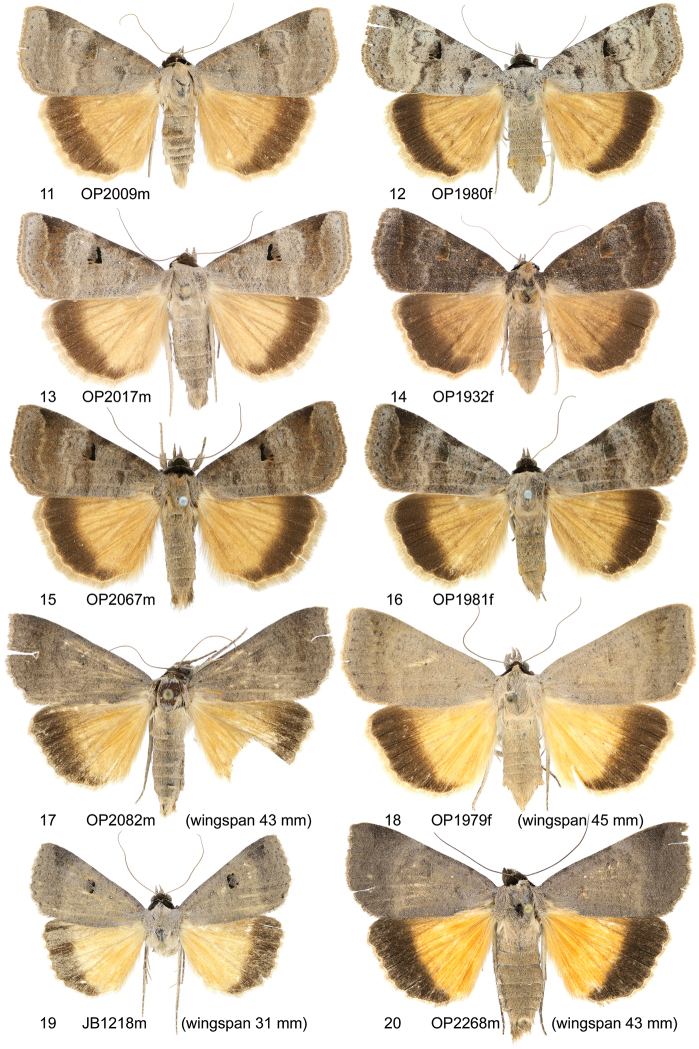
Adults. **11–16** *Lygephila kazachkaratavika* **11** neotype, ♂, Balamurum **12** ♀, Kazakhstan, Baigacum **13** ♂, Kazakhstan, Taldy-Kurgan reg. **14** ♀, Kazakstan, Baigacum **15** ♂, Kyrgyzstan, Issyk Kul **16** ♀, Kyrgyzstan, Issyk Kul **17, 18** *Lygephila lubrosa lubrosa* **17** neotype, ♂, Kazakhstan, Ili river **18** ♀, Kazakhstan, Baigacum **19, 20** *Lygephila lubrosa orbonaria* **19** neotype, ♂, Tajikistan, Pianj river **20** ♂, Tajikistan, Pianj river.

##### Destribution.

Kazakhstan, valley of the river Ili.

#### Lygephila lubrosa orbonaria

Taxon classificationAnimaliaLepidopteraErebidae

Stshetkin YuL & Stshetkin YuYu, 1994 [1997]

(TL: SW Tajikistan, “Tigrovaya balka” reserve) Figs 19 , 20

##### Type material examined.

**Neotype** (here designated) male, Tajikistan, down stream of Planj river, “Tigrovaya Balka” reserve, 1–5.08.2006, leg. V. Gurko, slide No. JB1218m (coll. P. Gyulai, will be deposited in HNHM Budapest).

##### Additional material examined.

1 ♂, S. Tajikistan, down stream of Pianj riv., “Tigrovaya Balka” reserve, 1–5.08.2006, V. Gurko lgt., slide No. OP2268m (coll. M. Dvořák).

##### Taxonomy.

Described as a subspecies of *Lygephila lubrosa*. The original description does not contain any information about the genitalia structures. However, the male genitalia show some recognisable differences compared with those of the nominate subspecies.

There is no trustworthy information about the holotype and paratypes of this taxon. According to the information from the Lepidoptera collection of IZIP, Stshetkins’s collection was totally destroyed sometime after the end of the 1990‘s. Also, there are no corresponding type labels in institute’s collection. To ensure the stability and identification of the taxon it is necessary to designate a neotype of *Lygephila lubrosa orbonaria*.

##### Diagnosis.

Differs from *Lygephila lubrosa lubrosa* by its smaller size and better marked reniform stigma. In the male genitalia, ssp. *orbonaria* differs from ssp. *lubrosa* by its narrower uncus without a medial thickening, and the narrower upper part of valva with more expressed asymmetry.

##### Description.

Wingspan 34–43 mm. The external features, with the exception of the smaller size and somewhat roundish forewing, match those of the nominate subspecies.

**Male genitalia** (Figs 35, 36). Uncus long, stout, sabre-like, anal tube membranous with oval hardening of tissue - scaphial crown on scaphium and sclerotized fig on subscaphium; valva elongated, wide, with parallel margins in the middle, tapering to apex; clasper digitiform, strong, thickened with wide base, placed subapically, somewhat asymmetrical, left one short, right one longer, almost reaches costa. Aedeagus tubular with narrow, long, acute sclerotised lamina on ventral side of carina. Vesica globular, multidiverticulate, membranous; basal diverticulum small; medial diverticulum large with small lateral hemispherical bulging; 1^st^ terminal diverticulum large, more or less wedge shaped, membranous with cauliflower-like part bearing numerous small pockets; 2^nd^ terminal diverticulum large, cone shaped, scobinated on top; 3^rd^ terminal diverticulum medial sized, bifurcated, Y-shaped; 4^th^ terminal diverticulum large, bilobate, located opposite to 3^rd^ medial diverticulum; terminal tube membranous, as long as aedeagus, opening point of terminal tube located near carina.

**Female genitalia.** Unknown.

**Figures 21–26.**
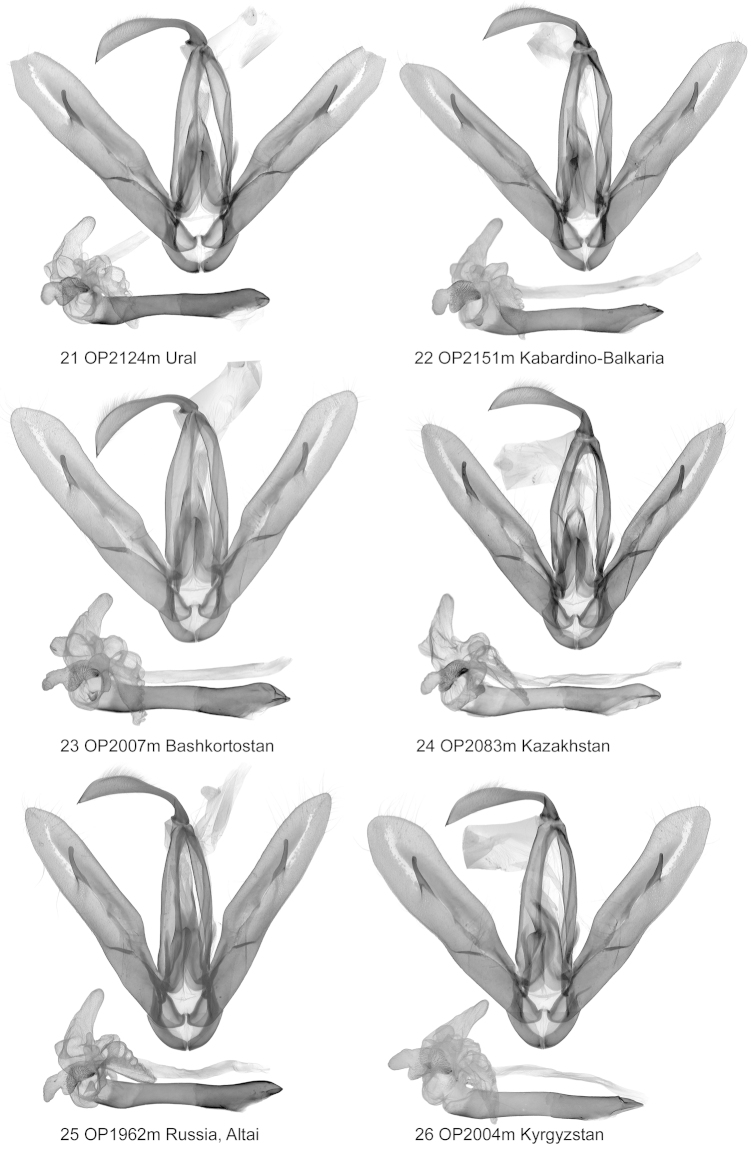
Clasping apparatus. *Lygephila lubrica*.

**Figures 27–32.**
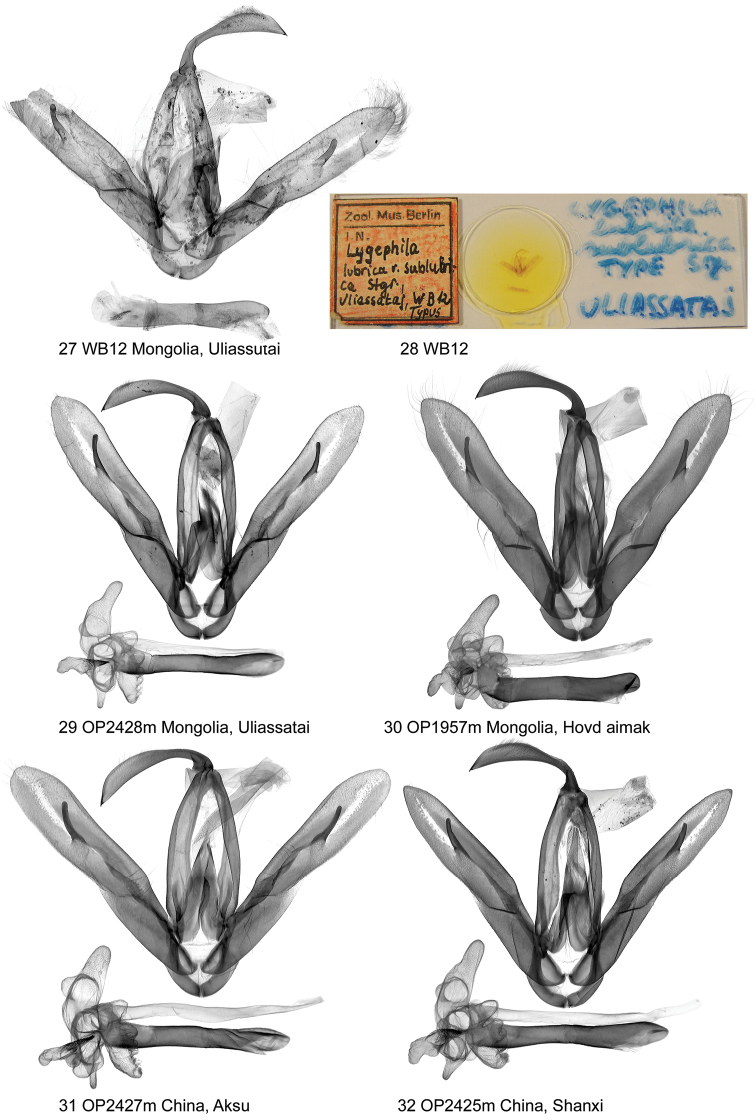
Clasping apparatus and genitalia slide. *Lygephila lubrica*.

**Figures 33–38.**
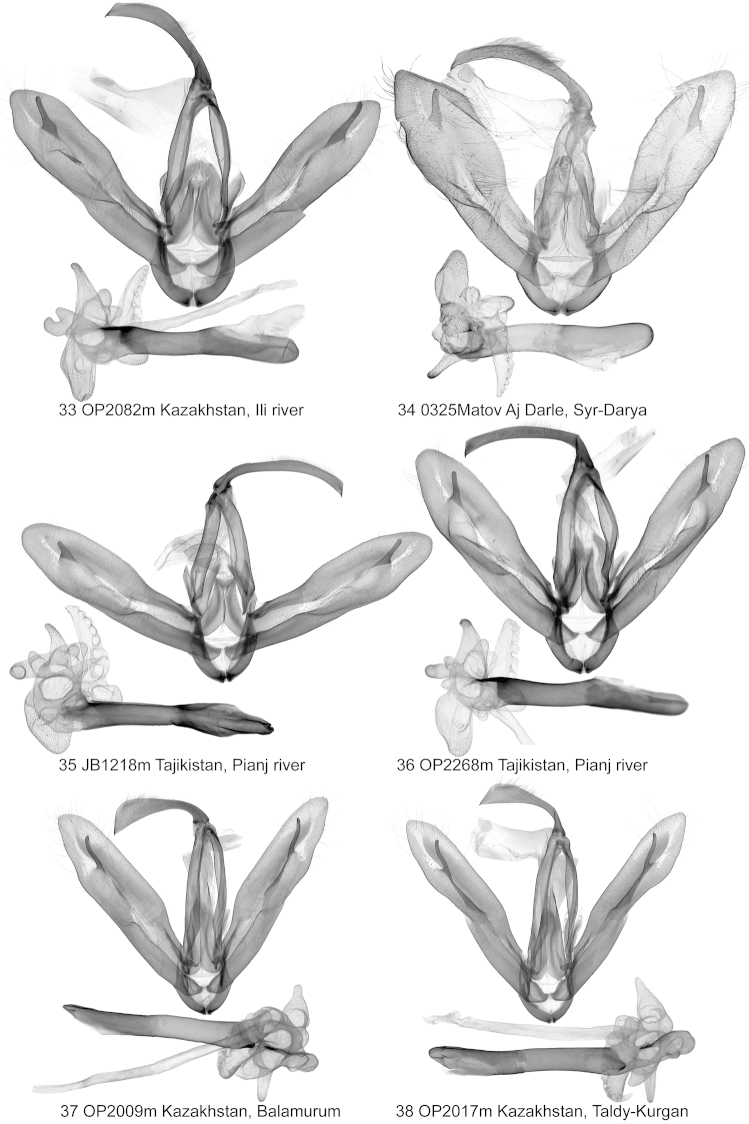
Clasping apparatus. **33, 34** *Lygephila lubrosa lubrosa* **33** neotype **35, 36** *Lygephila lubrosa orbonaria* **35** neotype **37, 38** *Lygephila kazachkaratavika* 37 neotype.

##### Distribution.

SW Tajikistan, Pianj river valley.

#### Lygephila kazachkaratavika

Taxon classificationAnimaliaLepidopteraErebidae

Stshetkin YuL & Stshetkin YuYu, 1994 [1997] stat. n.

(TL: Kazakhstan, Balamurum) Figs 11 –16

Lygephila lubrosa kazachkaratavika Stshetkin YuL & Stshetkin YuYu, 1994 [1997]

##### Type material examined.

**Neotype** (here designated) male (Fig. 11), 1 ♂, [Kazakhstan], Balamurum, Kara-tau, 1909.VI.21, leg. Koshantshikoff [Kozhantshikov], ex coll. John, slide No. OP2009m (coll. HNHM Budapest).

##### Additional material examined.

1 ♀, label1: [Kyrgyzstan], Asia Centr., (Issykul), 1896, revers label1: Toxocampa, von R. Tancré, 5.98, ex. coll. Püngeler, slide No. OP1981f (coll. MNHU); 1 ♂, [Kyrgyzstan], Issi-Kul, slide No. OP2067m (coll. NHMW); 1 ♀, label1: [Kazakhstan], Syr-Daria, Baigacum, Koshantschikoff, revers label1: 20.6.13, label2: 21/6, 1913, 3/7; 1 ♀, label1: [Kazakhstan], Syr-Daria, Baigacum, Koshantschikoff, revers label1: 21.6.13, label2: 21/6, 1913, 4/7; 1 ♀, label1: [Kazakhstan], Syr-Daria, Baigacum, Koshantschikoff, revers label1: 22.6.13, label2: 22/6, 1913, 5/7, ex. coll. Püngeler, slide No. OP1932f (coll. MNHU); 1 ♀, label1: [Kazakhstan], Syr-Daria, Baigacum, Koshantschikoff, revers label1: 23.VI.13, label2: 23/6, 1913, 6/7, ex. coll. Püngeler, slide No. OP1980f (coll. MNHU); 1 ♂, Kazakhstan, Taldy-Kurgan reg., Ili riv., Boroghudsir, 450m, 7–19.06.1996, slide No. OP2017m (coll. P. Gyulai).

##### Taxonomy.

Described as subspecies of *Lygephila lubrosa*. It is known that the author did not visit the museum collection of ZIN (ZISP) before writing his article on *Lygephila* and did not designate a holotype (personal comment of A. Matov). Also, potential type material of *Lygephila lubrosa kazachkaratavika* has not been found in any of the private collections where Stchetkin YuL’s material was purchased. So, the holotype most likely was never designated. To ensure stability of nomenclature and identification of the taxon it is necessary to designate neotype. A specimen from Kozhantshikov’s material preserved in the HNHM Budapest with the same label data as published in original description is hereby designated as neotype.

##### Diagnosis.

Easily separable from *Lygephila lubrica* and *Lygephila lubrosa* by the very contrasting, well-developed pattern on the forewings. In the male genitalia, it differs from all close relatives by its wider dilatation of the uncus, and the more acute apex of the valva (males); the longer antrum with a deep slit-like cleft on the posterior margin is diagnostic for females.

##### Description.

Wingspan 41–44 mm. Head and body brownish grey; collar dark chocolate brown. Forewing contrastingly marked, variable in coloration from mottled light greyish brown to uniform dark brown; noctuid pattern well developed; subbasal line traceable; antemedial line arched, consisting of three elongated patches edged by light fascia basally; medial shade waved, bifurcated from below cell to anal margin, with two costal patches; reniform stigma somewhat triangular, black, sometimes with satellite streak-like spots on outer margin; orbicular stigma absent; postmedial line distinct; subterminal line with light fascia; terminal line a row of black dots. Hindwing ochreous; transverse line distinct; small discal spot present on underside; border between pale proximal part and dark outer third diffuse; fringes ochreous.

**Male genitalia** (Figs 37, 38, 42, 43). Uncus stem short, stout, distal part dilated, terminated in fine tip; anal tube membranous with oval hardening of tissue - scaphial crown on scaphium and with sclerotized fig on subscaphium; valva elongated, relatively wide with parallel margins in the middle and convergent basally and distally; clasper digitiform, undulate, placed subapically, not reaching costa. Aedeagus straight, long, tubular, with small sclerotized fig on ventral side of carina. Vesica globular, multidiverticulate, membranous; basal diverticulum small; medial diverticulum large, cupola shaped with two hemispherical chambers medially and basally; 1^st^ terminal diverticulum large, more or less wedge shaped, with one part densely scobinated and membranous cauliflower-like, opposite part bearing numerous small pockets; 2^nd^ terminal diverticulum tubular, elongated, scobinated on top; 3^rd^ terminal diverticulum medium sized with lateral bulging; 4^th^ terminal diverticulum large, conical, situated opposite to 3^rd^ medial diverticulum; terminal tube membranous, as long as aedeagus, opening point of terminal tube located subbasally near carina.

**Female genitalia** (Figs 62, 63). Ovipositor relatively large, broad, papillae anales hairy with very long setae on apical edges. Apophyses anteriores long and thin, apophyses posteriores thin, somewhat longer than apophyses anteriores. Ostium broad, antrum tapering, funnel shaped, posterior margin deeply incised producing slit-like cleft; ductus bursae small; appendix bursae small; corpus bursae membranous, ovoid.

**Figures 39–41.**
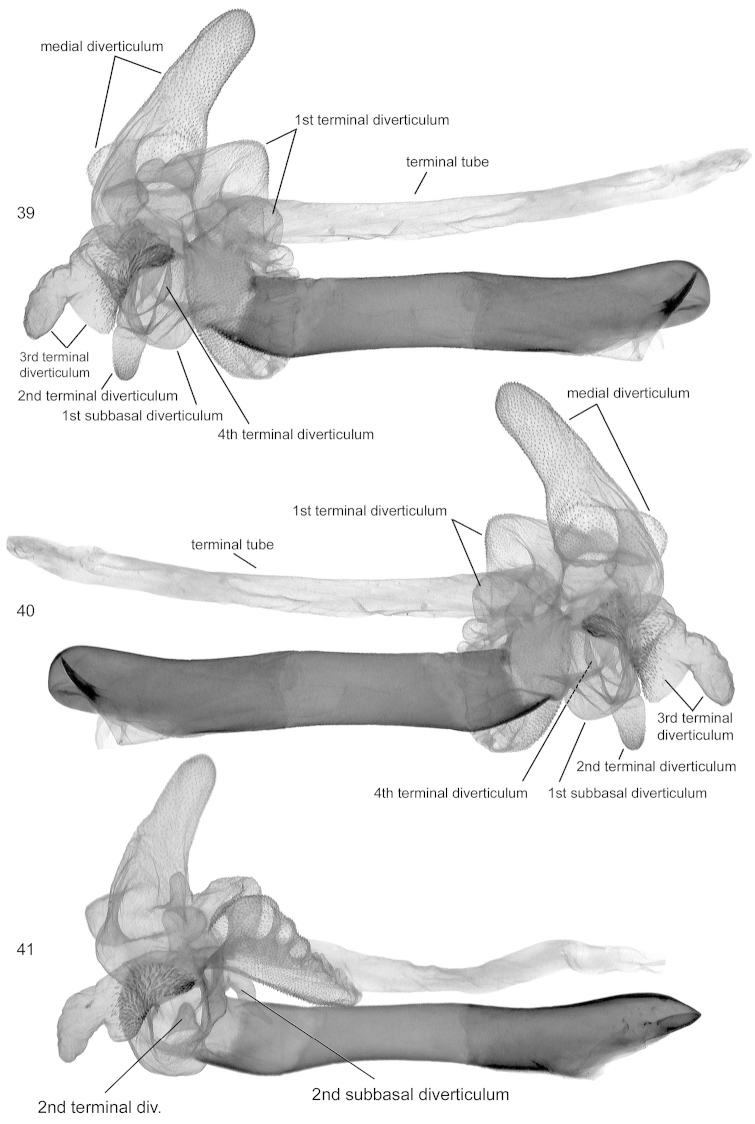
Vesica structure of *Lygephila lubrica*. **39, 40** Mongolia, Hovd aimak, slide No. OP1957m **39** lateral view **40** lateral view opposite side **41** Russia, Altai, Kupchegen, slide No. OP1962m, lateral view.

**Figures 42–45.**
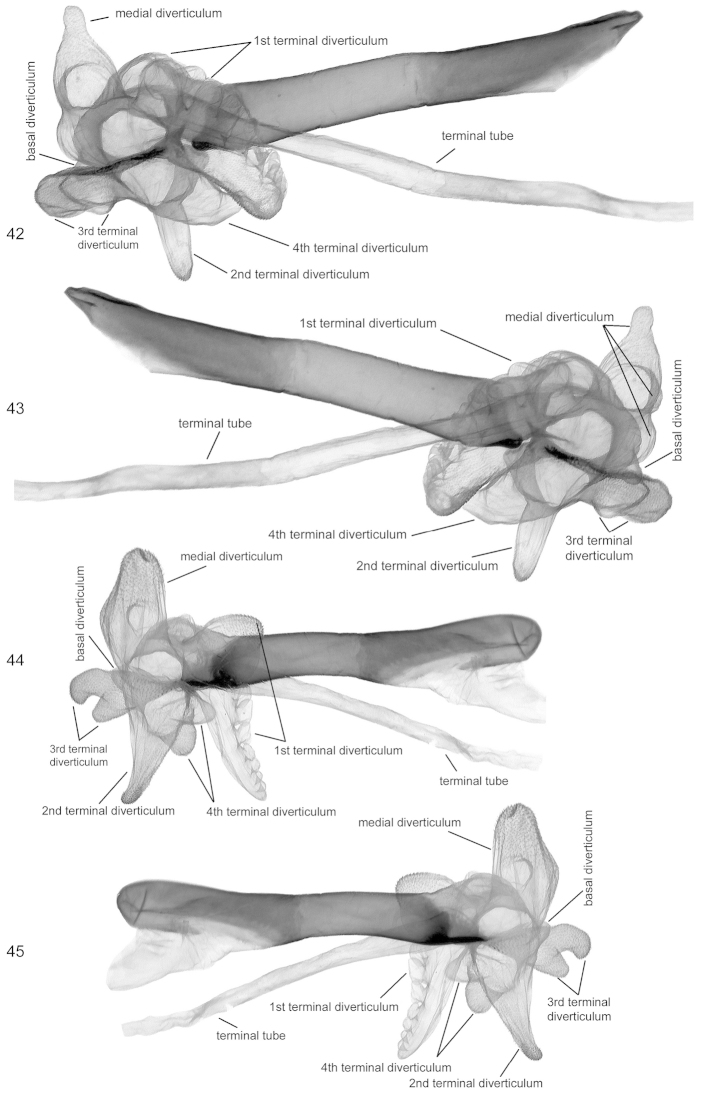
Vesica structure. **42, 43** *Lygephila kazachkaratavika*, neotype, Kazakhstan, Balamurum, slide No. OP2009m **42** lateral view **43** lateral view opposite side **44, 45** *Lygephila lubrosa lubrosa*, neotype, Kazakhstan, Ili river, slide No. OP2082m **44** lateral view **45** lateral view opposite side.

**Figures 46–63.**
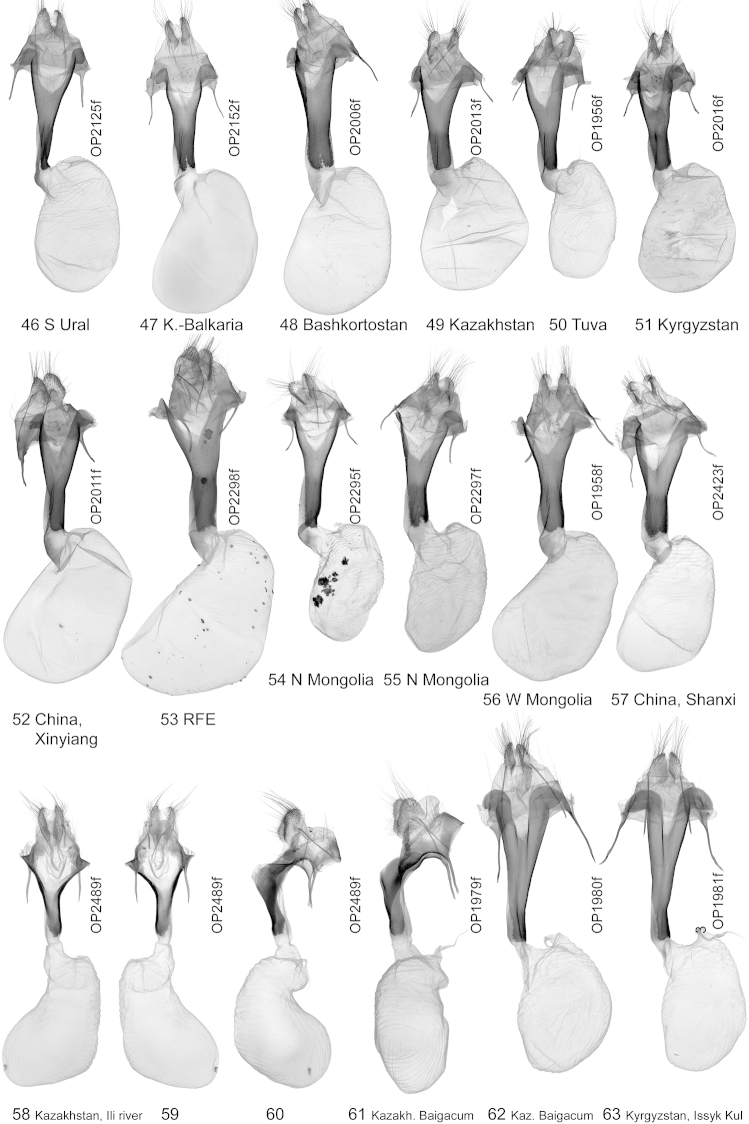
Female genitalia. **46–57** *Lygephila lubrica* **58–61** *Lygephila lubrosa lubrosa* **58** ventral view **59** dorsal view **60** lateral view **61** lateral view **62, 63** *Lygephila kazachkaratavika*.

##### Distribution.

Kazakhstan, Kyrgyzstan.

#### Lygephila lupina

Taxon classificationAnimaliaLepidopteraErebidae

(Graeser, 1890) stat. n.

Figs 64 , 65

Toxocampa lupina Graeser, 1890, Berliner Entomologische Zeitschrift, 35: 71–84. (TL: [Russia, Judish Autonomy, Radde] Raddefka)
Lygephila mirabilis (Bryk, 1948), **syn. n.**
Eccrita mirabilis Bryk, 1948 (TL: N Korea, Shuotsu)

##### Type material examined.

♀ Type, Amur Centr. (Radde), [18]87, ex. coll. Püngeler, slide No. OP1931f (coll. MNHU).

##### Additional material examined.

1 ♀, [RFE], Ussuriysk dist., Kajmanovka, 20.VII.[19]82, leg. Ivanov, slide No. 0321Matov (coll. ZISP). 1 ♀, [China], Tapaishan im Tsinling, Sued-Shensi, Ca. 1700 m, 14.7.1936, H. Höne, slide No. OP2402f, 1 ♀, [China], Tapaishan im Tsinling, Sued-Shensi, Ca. 1700 m, 10.8.1936, H. Höne, slide No. OP2403f (coll. ZFMK).

##### Note.

There is a lot of confusion between *Lygephila mirabilis* and *Lygephila vulcanea* (Butler, 1881) in the literature with regard to illustrations of the adults and genitalia. The taxonomic clarification of this problem will be given in a separate publication.

##### Taxonomy.

**Figures 64–69.**
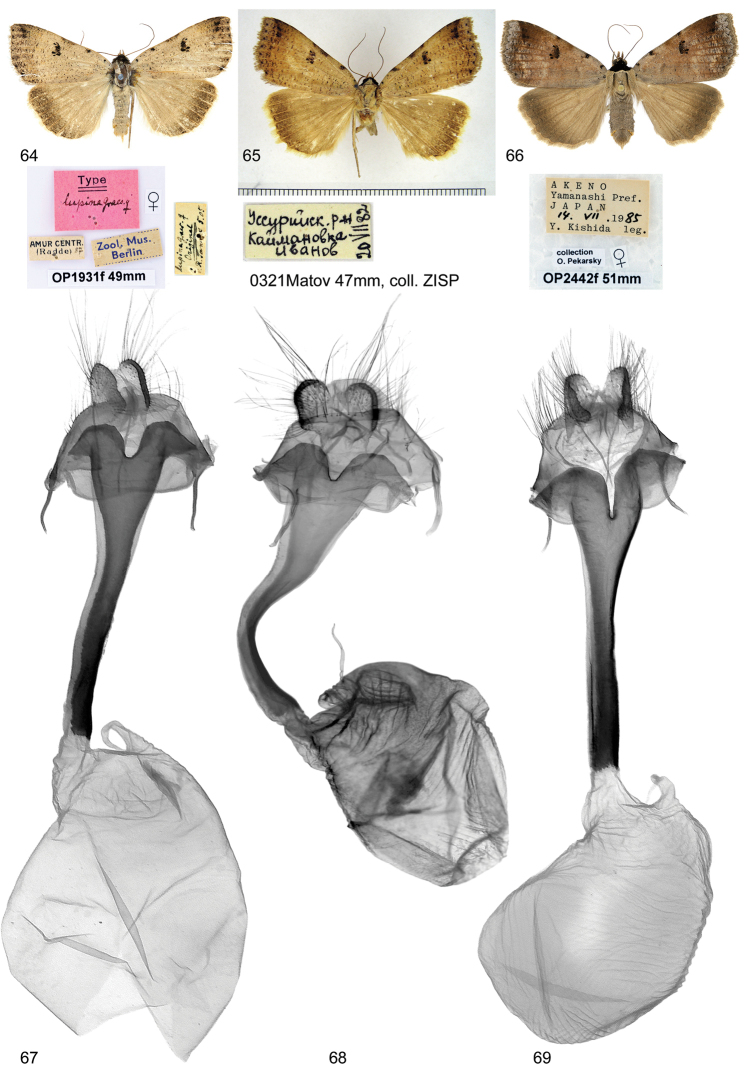
**64–66** Adults. 64, 65 *Lygephila lupina* (=*mirabilis*) **64** ♀, Type, Russia, Raddefka **65** Russia, Kajmanovka **66** *Lygephila vulcanea* ♀, Japan **67–69** Female genitalia **67, 68** *Lygephila lupina* (=*mirabilis*) 67 Russia, Raddefka, slide No. OP1931f **68** Russia, Kajmanovka, slide No. 0321Matov **69** *Lygephila vulcanea*, Japan, slide No. OP2442f.

The identity of *Lygephila lupina* was unclear for a long time. *Lygephila lupina* was described, according to the original description, from Radde, central Amur [Khabarovsk region] (Graeser 1890) on the basis of a single male from the collection of Taylor Tancré, in comparison with *Lygephila maxima* (Bremer, 1861). The Püngeler collection, now in MNHU, contains a female specimen with a type label (Fig. 64). One can be convinced from the information given by the handwriting of Püngeler on the opposite side of the collecting label (made in May 1905) that this is a true type specimen from the Tancré collection and it is a female, not a male. Thus, Graeser was mistaken about the sex of the type specimen. The study of the genitalia of the type specimen reveals that *Lygephila lupina* is conspecific with the species known as *Lygephila mirabilis* (Bryk, 1948). *Lygephila mirabilis*, therefore, is a junior synonym of *Lygephila lupina*, syn. n.

##### Diagnosis.

The distinctive features of *Lygephila lupina* and *Lygephila vulcanea* (Fig. 66) are given in the works of Sviridov (1990) and Kononenko (1996) (under the names *Lygephila vulcanea* and *Lygephila mirabilis*). The main external differences between the two species are found in the colouration and shape of the forewing: *Lygephila lupina* is broader winged and paler in colouration, usually ochreous brown to buff coloured, whereas *Lygephila vulcanea* is darker, deep brown to claret brown, most often with a clearly visible violaceous shade and the forewing apex is somewhat more pointed. In the majority of the specimens the reniform stigma of *Lygephila lupina* is stronger, sharper, and more distinctly marked against the paler background. The female genitalia differ from those of *Lygephila vulcanea* (Fig. 69) by the shallower incision of the posterior margin of the antrum.

##### Description.

Wingspan 44–49 mm. Head and body brownish grey; collar dark chocolate brown. Forewing brownish grey with sparse dark brown irroration; subbasal line indistinct; antemedial line arched with costal patch; reniform stigma large, dark brown, consists of 5 or 6 streak-like spots; orbicular stigma absent; postmedial and subterminal lines distinct; terminal line a row of black dots. Hindwing brownish; small discal spot present on underside; outer third dark brown; fringes as ground color.

**Female genitalia** (Figs 67, 68). Ovipositor long, papillae anales large, hairy with long setae on apical edges. Apophyses anteriores relatively short, apophyses posteriores thin, longer than apophyses anteriores. Antrum long, narrow anteriorly, dilated posteriorly, ostium broad, posterior margin with small U-shaped cleft. Corpus bursae membranous, ovoid.

##### Distribution.

Russian Far East, China, Korea.

## Supplementary Material

XML Treatment for Lygephila lubrica lubrica

XML Treatment for Lygephila lubrosa lubrosa

XML Treatment for Lygephila lubrosa orbonaria

XML Treatment for Lygephila kazachkaratavika

XML Treatment for Lygephila lupina
